# Supplementary material for: An elusive electron shuttle from a facultative anaerobe
Source: eLife. 2019 Jun 24;8:e48054. doi: 10.7554/eLife.48054 (PMC6687433; doi:10.7554/eLife.48054)
Supplement: Supplementary file 1. — (A) Acquisition of non-E. coli CGCS bacterial strains. (B) E. coli strains purchased from Coli Genetic Stock Center. [file elife-48054-supp1.docx]

**Supporting Information**

**An Elusive Electron Shuttle from a Facultative Anaerobe**

Emily Mevers^1†^, Lin Su^2,3†^, Gleb Pischany^1,4^, Moshe Baruch^2^, Jose Cornejo^2^, Elissa Hobert^1^, Eric Demise^1^, Caroline M. Ajo-Franklin^2,5*^, & Jon Clardy^1*^

Departments of ^1^Biological Chemistry and Molecular Pharmacology, and ^4^Microbiology and Immunobiology, Harvard Medical School, Boston, MA 02115, USA

^2^Molecular Foundry Division, and ^5^Molecular Biophysics and Integrated Bioimaging Division, Lawrence Berkeley National Laboratory, University of California, Berkeley, CA 94720, USA

^3^State Key Laboratory of Bioelectronics, School of Biological Science and Medical Engineering, Southeast University, Nanjing, 210018, China

^†^These authors contributed equally to this work

**Table of Contents:**

Supplementary file 1A: Acquisition of non-*E. coli* bacterial strains…………………………………………………………………………………………………………………………..……………...pg. 2

Supplementary file 1B: *E. coli* strains purchased from Coli Genetic Stock Center……………………………………………………………………………………………………………………..…..……………….pg. 2

References………………………………………………………………………………………………………………………………......pg. 3

| **Table S1.** Acquisition of non-*E. coli* CGCS bacterial strains | | | |
| --- | --- | --- | --- |
| **Strain** | **Acquired from** | **Commercial ID** |  |
| *S. oneidensis* MR-1 | Kolter lab [(Newman and Kolter, 2000)](https://paperpile.com/c/4tWkDK/5PPr) | **n/a** |  |
| *S. oneidensis* *menC::Tn10* | Kolter lab [(Newman and Kolter, 2000)](https://paperpile.com/c/4tWkDK/5PPr) | **n/a** |  |
| *S. oneidensis menA::Himar* | Barstow lab [(Baym et al., 2016)](https://paperpile.com/c/4tWkDK/g5A9) | **n/a** |  |
| *V. cholerae* V52 | Kolter lab | **n/a** |  |
| *B. fragilis* | ATCC | 25285 |  |
| *L. lactis lactis* | DSM | 20481 |  |
| *S. oneidensis* Δ*bfe* | Gralnick lab [(Kotloski and Gralnick, 2013)](https://paperpile.com/c/4tWkDK/KhY8l) | **n/a** |  |
| *S. oneidensis* Δ*mtr* | Gralnick lab [(Coursolle and Gralnick, 2012)](https://paperpile.com/c/4tWkDK/kwnw) | **n/a** |  |

| **Table S2.** *E. coli* strains purchased from Coli Genetic Stock Center | | | |
| --- | --- | --- | --- |
| **Strain** | **Name** | **CGSC#** | **Genotype** |
| Parent | BW25113 | 7636 | F-, *Δ(araD-araB)567*, *ΔlacZ4787*(::rrnB-3), *λ^-^*, *rph-1*, *Δ(rhaD-rhaB)568*, *hsdR514* |
| *ΔaspC* | JW0911-1 | 8924 | F-, Δ(araD-araB)567, ΔlacZ4787(::rrnB-3), λ-, ΔaspC745::kan, rph-1, Δ(rhaD-rhaB)568, hsdR514 |
| *ΔtyrB* | JW4014-2 | 10886 | F-, Δ(araD-araB)567, ΔlacZ4787(::rrnB-3), λ-, rph-1, Δ(rhaD-rhaB)568, ΔtyrB747::kan, hsdR514 |
| *ΔargD* | JW3322-1 | 10482 | F-, Δ(araD-araB)567, ΔlacZ4787(::rrnB-3), λ-, ΔargD767::kan, rph-1, Δ(rhaD-rhaB)568, hsdR514 |
| *ΔgabT* | JW2637-4 | 11775 | F-, Δ(araD-araB)567, ΔlacZ4787(::rrnB-3), λ-, ΔgabT743::kan, rph-1, Δ(rhaD-rhaB)568, hsdR514 |
| *ΔserC* | JW0890-1 | 8913 | F-, Δ(araD-araB)567, ΔlacZ4787(::rrnB-3), λ-, ΔserC731::kan, rph-1, Δ(rhaD-rhaB)568, hsdR514 |
| *ΔilvE* | JW5606-1 | 11592 | F-, Δ(araD-araB)567, ΔlacZ4787(::rrnB-3), λ-, rph-1, ΔilvE721::kan, Δ(rhaD-rhaB)568, hsdR514 |
| *ΔguaA* | JW2491-1 | 11876 | F-, Δ(araD-araB)567, ΔlacZ4787(::rrnB-3), λ-, ΔguaA756::kan, rph-1, Δ(rhaD-rhaB)568, hsdR514 |
| *ΔavtA* | JW5652-1 | 11634 | F-, Δ(araD-araB)567, ΔlacZ4787(::rrnB-3), λ-, ΔavtA755::kan, rph-1, Δ(rhaD-rhaB)568, hsdR514 |
| *ΔarnB* | JW5372-1 | 11347 | F-, Δ(araD-araB)567, ΔlacZ4787(::rrnB-3), λ-, ΔarnB734::kan, rph-1, Δ(rhaD-rhaB)568, hsdR514 |
| *ΔhisC* | JW2003-1 | 9649 | F-, Δ(araD-araB)567, ΔlacZ4787(::rrnB-3), λ-, ΔhisC790::kan, rph-1, Δ(rhaD-rhaB)568, hsdR514 |
| *ΔybdL* | JW0593-3 | 8709 | F-, Δ(araD-araB)567, ΔlacZ4787(::rrnB-3), ΔybdL739::kan, λ-, rph-1, Δ(rhaD-rhaB)568, hsdR514 |
| *ΔpuuE* | JW1295-1 | 11635 | F-, Δ(araD-araB)567, ΔlacZ4787(::rrnB-3), λ-, ΔpuuE738::kan, rph-1, Δ(rhaD-rhaB)568, hsdR514 |
| *ΔyjiR* | JW4303-1 | 11079 | F-, Δ(araD-araB)567, ΔlacZ4787(::rrnB-3), λ-, rph-1, Δ(rhaD-rhaB)568, ΔyjiR739::kan, hsdR514 |
| *ΔydcR* | JW1434-1 | 9254 | F-, Δ(araD-araB)567, ΔlacZ4787(::rrnB-3), λ-, ΔydcR730::kan, rph-1, Δ(rhaD-rhaB)568, hsdR514 |
| *ΔyhfS* | JW3339-1 | 10494 | F-, Δ(araD-araB)567, ΔlacZ4787(::rrnB-3), λ-, ΔyhfS783::kan, rph-1, Δ(rhaD-rhaB)568, hsdR514 |
| *ΔastC* | JW1737-4 | 11761 | F-, Δ(araD-araB)567, ΔlacZ4787(::rrnB-3), λ-, ΔastC746::kan, rph-1, Δ(rhaD-rhaB)568, hsdR514 |
| *ΔpatA* | JW5510-1 | 11435 | F-, Δ(araD-araB)567, ΔlacZ4787(::rrnB-3), λ-, ΔygjG763::kan, rph-1, Δ(rhaD-rhaB)568, hsdR514 |
| *ΔyfdZ* | JW2376-1 | 9898 | F-, Δ(araD-araB)567, ΔlacZ4787(::rrnB-3), λ-, ΔyfdZ788::kan, rph-1, Δ(rhaD-rhaB)568, hsdR514 |
| *ΔyfbQ* | JW2287-1 | 9839 | F-, Δ(araD-araB)567, ΔlacZ4787(::rrnB-3), λ-, ΔyfbQ772::kan, rph-1, Δ(rhaD-rhaB)568, hsdR514 |
| *ΔglmS* | E111 | 5393 | F-, Δ(gpt-proA)62, lacY1, tsx-33, galE28(GalS), λ-, rpsL31(strR), kdgK51, xylA5, mtl-1, glmS1, argE3(Oc), thiE1 |
| *ΔhemL* | GE1376 | 7264 | Hfr(PO12), thr-1, leuB6(Am), panD2, hemL206, lacZ4, glnX44(AS), λ-, rfbC1, rpsL8, thiE1 |
| *ΔbioA* | JW0757-1 | 8816 | F-, Δ(araD-araB)567, ΔlacZ4787(::rrnB-3), λ-, ΔbioA746::kan, rph-1, Δ(rhaD-rhaB)568, hsdR514 |
| *ΔmenA* | JW3901-1 | 10816 | F-, Δ(araD-araB)567, ΔlacZ4787(::rrnB-3), λ-, rph-1, Δ(rhaD-rhaB)568, ΔmenA789::kan, hsdR514 |
| *ΔmenC* | JW2256-1 | 9819 | F-, Δ(araD-araB)567, ΔlacZ4787(::rrnB-3), λ-, ΔmenC743::kan, rph-1, Δ(rhaD-rhaB)568, hsdR514 |
| *ΔmenB* | JW2257-2 | 11787 | F-, Δ(araD-araB)567, ΔlacZ4787(::rrnB-3), λ-, ΔmenB744::kan, rph-1, Δ(rhaD-rhaB)568, hsdR514 |

References

[Baym M, Shaket L, Anzai IA, Adesina O, Barstow B. 2016. Rapid construction of a whole-genome transposon insertion collection for Shewanella oneidensis by Knockout Sudoku. *Nature Communications*. doi:](http://paperpile.com/b/4tWkDK/g5A9)[10.1038/ncomms13270](http://dx.doi.org/10.1038/ncomms13270)

[Coursolle D, Gralnick JA. 2012. Reconstruction of Extracellular Respiratory Pathways for Iron(III) Reduction in Shewanella Oneidensis Strain MR-1. *Frontiers in Microbiology*. doi:](http://paperpile.com/b/4tWkDK/kwnw)[10.3389/fmicb.2012.00056](http://dx.doi.org/10.3389/fmicb.2012.00056)

[Kotloski NJ, Gralnick JA. 2013. Flavin electron shuttles dominate extracellular electron transfer by Shewanella oneidensis, mBio, 4, e00553--12; doi: 10.1128/mBio. 00553-12. *Chapter 1: General introduction & Objectives*.](http://paperpile.com/b/4tWkDK/KhY8l)

[Newman DK, Kolter R. 2000. A role for excreted quinones in extracellular electron transfer. *Nature* **405**:94–97.](http://paperpile.com/b/4tWkDK/5PPr)
