# Supplementary material for: An elusive electron shuttle from a facultative anaerobe
Source: eLife. 2019 Jun 24;8:e48054. doi: 10.7554/eLife.48054 (PMC6687433; doi:10.7554/eLife.48054)

ANOVAOneWay (2/22/2019 11:17:32)   Note: the ΔmenC in this report is menC::Tn10

Notes

Description	Perform One-Way ANOVA
User Name	su2li
Operation Time	2/22/2019 11:17:32
Report Status	Report generated from Data Changed
Data Filter	No

Input Data

	Data	Range
MR-1	[Book4]Sheet1!A"MR-1"	[1*:3*]
ΔmenC	[Book4]Sheet1!B"menC::tn"	[1*:3*]
MR-1+ACNQ	[Book4]Sheet1!C"MR-1+ACNQ"	[1*:3*]
ΔmenC+ACN	[Book4]Sheet1!D"menC::tn+ACN	[1*:3*]

Descriptive Statistics

	N Analysis	N Missing	Mean	Standard Deviation	SE of Mean
MR-1	3	0	1.38543	0.23027	0.13295
menC::tn	3	0	1.14818	0.24799	0.14318
MR-1+ACNQ	3	0	46.67687	3.35527	1.93717
menC::tn+AC	3	0	42.37444	4.83352	2.79063

One Way ANOVA

Overall ANOVA

	DF	Sum of Squares	Mean Square	F Value	Prob>F
Model	3	5641.83491	1880.61164	216.56535	5.36088E-8
Error	8	69.47045	8.68381		
Total	11	5711.30536			

Null Hypothesis: The means of all levels are equal.
Alternative Hypothesis: The means of one or more levels are different.
At the 0.001 level, the population means are significantly different.

Fit Statistics

	R-Square	Coeff Var	Root MSE	Data Mean
	0.98784	0.1287	2.94683	22.89623

Means Comparisons

Tukey Test

	MeanDiff	SEM	q Value	Prob	Alpha	Sig	LCL	UCL
ΔmenC MR-1	-0.23725	2.40608	0.13945	0.99963	0.001	0	-15.5107	15.0362
MR-1+ACNQ MR-1	45.29144	2.40608	26.62083	1.84137E-7	0.001	1	30.01799	60.56489
MR-1+ACNQ ΔmenC	45.52869	2.40608	26.76028	1.71633E-7	0.001	1	30.25524	60.80214
ΔmenC+ACNQ MR-1	40.98901	2.40608	24.092	5.34533E-7	0.001	1	25.71556	56.26246
ΔmenC+ACNQ ΔmenC	41.22626	2.40608	24.23145	5.05081E-7	0.001	1	25.95281	56.49971
ΔmenC+ACNQ MR-1+A	-4.30243	2.40608	2.52883	0.34463	0.001	0	-19.57588	10.97102

Sig equals 1 indicates that the difference of the means is significant at the 0.001 level.
Sig equals 0 indicates that the difference of the means is not significant at the 0.001 level.

Homogeneity of Variance Test

Levene's Test(Absolute Deviations)

	DF	Sum of Squares	Mean Square	F Value	Prob>F
Model	3	26.82522	8.94174	6.22707	0.01733
Error	8	11.48758	1.43595		

At the 0.001 level, the population variances are not significantly different.

Powers

	Alpha	Sample Size	Power
Actual Power	0.001	12	1

Box Charts

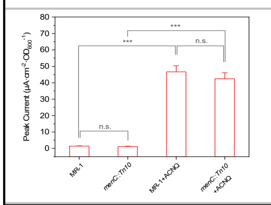

Supplement: Supplementary file 2. [file elife-48054-supp2.pdf]
